# Head and neck cancer patients with geriatric deficits are more often non-responders and lost from follow-up in quality of life studies

**DOI:** 10.1007/s00405-024-08528-w

**Published:** 2024-03-01

**Authors:** Julius de Vries, Dannie J. Vermue, Grigory Sidorenkov, Suzanne Festen, Johannes A. Langendijk, Geertruida H. de Bock, Gyorgy B. Halmos

**Affiliations:** 1 Department of Otorhinolaryngology, Head and Neck Surgery, University Medical Center Groningen, University of Groningen, Hanzeplein 1, P.O. Box 30 001, 9700 RB Groningen, The Netherlands; 2 Department of Epidemiology, University Medical Center Groningen, University of Groningen, Groningen, The Netherlands; 3 Department of Geriatric Medicine, University Medical Center Groningen, University of Groningen, Groningen, The Netherlands; 4 Department of Radiation Oncology, University Medical Center Groningen, University of Groningen, Groningen, The Netherlands

**Keywords:** Bias, Geriatric deficit, Head and neck cancer, Lost from follow-up, Non-response to questionnaires, Patient-reported outcome measures

## Abstract

**Objectives:**

To identify associations between frailty and non-response to follow-up questionnaires, in a longitudinal head and neck cancer (HNC) study with patient-reported outcome measures (PROMs).

**Materials and methods:**

Patients referred with HNC were included in OncoLifeS, a prospective data-biobank, underwent Geriatric Assessment (GA) and frailty screening ahead of treatment, and were followed up at 3, 6, 12 and 24 months after treatment using the European Organisation for Research and Treatment of Cancer Quality of Life Questionnaire Core 30 and Head and Neck 35. Statistical analysis for factors associated with non-response was done using Generalized Linear Mixed Models.

**Results:**

289 patients were eligible for analysis. Mean age was 68.4 years and 68.5% were male. Restrictions in Activities of Daily Living [OR 4.46 (2.04–9.78)] and Instrumental Activities of Daily Living [OR 4.33 (2.27–8.24)], impaired mobility on Timed Up and Go test [OR 3.95 (1.85–8.45)], cognitive decline [OR 4.85 (2.28–10.35)] and assisted living (OR 5.54 (2.63–11.67)] were significantly associated with non-response. Frailty screening, with Geriatric 8 and Groningen Frailty Indicator, was also associated with non-response [OR, respectively, 2.64 (1.51–4.59) and 2.52 (1.44–4.44)]. All findings remained significant when adjusted for other factors that were significantly associated with non-response, such as higher age, longer study duration and subsequent death.

**Conclusion:**

Frail HNC patients respond significantly worse to follow-up PROMs. The drop-out and underrepresentation of frail patients in studies may lead to attrition bias, and as a result underestimating the effect sizes of associations. This is of importance when handling and interpreting such data.

## Introduction

The global incidence of cancer is rapidly increasing, specifically among older populations [1]. Older patients, however, are strongly underrepresented in clinical trials in all fields of medicine [2]. This is the case for large cancer trials, which are important for the establishment of international guidelines, as well [3–5]. Barriers for trial inclusion can be raised by the system, by care-providers, but also by patients themselves [6].

Besides the evident difficulty of including older patients in clinical studies, retaining older patients in clinical studies may be difficult as well, and lead to higher non-response and study drop-out [7, 8]. This may be referred to as ‘attrition’. Especially with the growing use of patient-reported outcome measures (PROM’s), the risk of non-response is lurking, and this may be even more the case in the older and frail population [9]. PROM’s, however, such as questionnaires for quality of life (QoL), are increasingly being recognized as important outcome measures, besides recurrence or survival alone. Specifically for older patients this may be the case, as they may prioritize outcomes such as QoL over length of life, for example [10].

Yet, the occurrence of non-response and study drop-out for older and frail patients relative to their younger and fit counterparts is important to know. Systematic loss of patients from specific study groups may lead to attrition bias [11]. Consequences of this may be under- or overestimating outcomes, misinterpretation of the results and poor generalizability.

The age of patients with head and neck cancer averages around 65 and the burden of geriatric deficits and therewith frailty is large in this population, compared to patients with other solid malignancies [12]. The risk of introducing bias into studies may therefore be high. In our previous studies we encountered that frail patients were more difficult to include because of their poor response to baseline questionnaires [13]. Therefore, the goal of the current study was to investigate whether frail patients exhibit more non-response than non-frail patients to follow-up questionnaires and whether specific items of a routinely performed geriatric assessment (GA) are associated with non-response.

## Materials and methods

### Study design

This study covers a retrospective analysis of prospectively collected data from the longitudinal observational Oncological Life Study (OncoLifeS), a large hospital-based oncological data-biobank at the University Medical Center Groningen (UMCG), Groningen, The Netherlands [14]. OncoLifeS is approved by the Institutional Review board of the UMCG, this study was approved by the scientific committee of OncoLifeS. In OncoLifeS patients are included after providing written informed consent. Between October 2014 and May 2015, all patients referred with (suspicion of) primary or recurrent cancer in the head and neck area (mucosal, salivary gland and cutaneous) were consecutively included. Patients were seen at the outpatient clinic of the departments of Otorhinolaryngology, Head and Neck Surgery, Oral and Maxillofacial Surgery and Radiation Oncology. Patients underwent a GA, including frailty screening, at baseline, before treatment. Patients were excluded from the analysis when initially palliative or non-standard treatment was conducted or when patients did not return the baseline questionnaires. Also, data of patients were excluded when recurrence or death occurred during follow-up, from that moment onward. Patients were followed up during 2 years after treatment using QoL questionnaires (*see* Follow-up).

### Patient, tumour and treatment characteristics

Patient, tumour and treatment characteristics were withdrawn from the OncoLifeS data-biobank. Disease was staged according to the seventh edition of the Union for International Cancer Control’s TNM Classification [15].

### Baseline assessments

Before treatment patients underwent GA, including a frailty screening and assessment of the somatic, functional, psychological and socio-environmental domains. Somatic assessments included scoring of the 27-item Adult Comorbidity Evaluation (ACE-27), polypharmacy (5 or more medications) and the Malnutrition Universal Screening Tool (MUST) [18–20]. Functional assessments were Activities of Daily Living (ADL), Instrumental Activities of Daily Living (IADL) and the Timed Up & Go (TUG) with a cut-off at 13.5s [21–24]. The Mini Mental State Examination (MMSE) and the 15-item Geriatric Depression Scale (GDS-15) were used for the psychological assessments [25–27]. Marital status, living situation and educational level assessed for the socio-environmental domain and were registered as part of a standardized questionnaire. Frailty screening consisted of the Groningen Frailty Indicator (GFI) and Geriatric 8 (G8) questionnaires [16, 17].

### Follow-up

Patients were followed up using the European Organisation for Research and Treatment of Cancer Quality of Life Questionnaire Core 30 (EORTC QLQ-C30) and Head and Neck 35 (EORTC-QLQ-HN35) at 3, 6, 12 and 24 months after treatment. Follow-up was conducted by sending and returning questionnaires by mail (dept. of Otorhinolaryngology, Head and Neck Surgery, dept. of Oral and Maxillofacial Surgery) or by filling out questionnaires at the outpatient clinic (dept. of Radiation Oncology). This difference between methods was incorporated as a variable in the dataset.

### Outcome

Non-response was defined as both complete QoL questionnaires missing in the dataset. This was recalculated to a binary outcome (yes/no) for each of the follow-up moments (3, 6, 12 and 24 months) after treatment initiation, regardless of the previous outcomes, and until recurrence or death occurred.

### Statistical analysis

All statistical procedures were performed with SPSS Statistics 28 (IBM). Descriptive statistics are presented as *n* (%) unless specified otherwise. Generalized Linear Mixed Models (GLMM) were used to calculate odds ratios of the association between frailty and non-response for any data point in the follow-up. As an advantage, this allows for using all data points before exclusion due to death or recurrence and thus reducing risk of bias. Patients with upcoming (but not yet diagnosed) recurrence or death may have worse response; therefore, ‘subsequent recurrence’ and ‘subsequent death’ tested as variables as well. For all models, non-response was the target variable in a binary logistic fashion. For fixed effects an intercept and the predictor variable were included. For random effects an intercept was included and covariance type was set to unstructured. At first, GLMMs were carried out for patient characteristics, both univariate and in a multivariable model. Second, frailty screening instruments and GA items were evaluated in a GLMM, both in an unadjusted model and then in a model adjusted for all relevant patient characteristics.

## Results

**Fig. 1:**
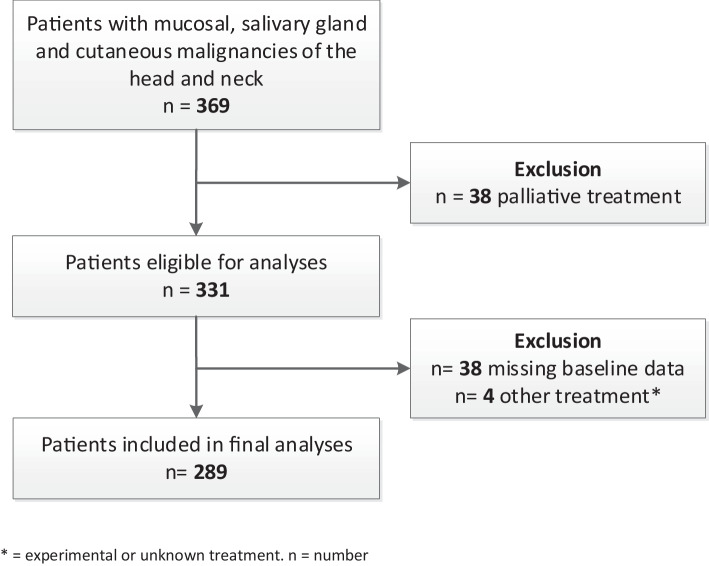
Flowchart of study inclusion. * = experimental or unknown treatment. *n* = number

**Fig. 2:**
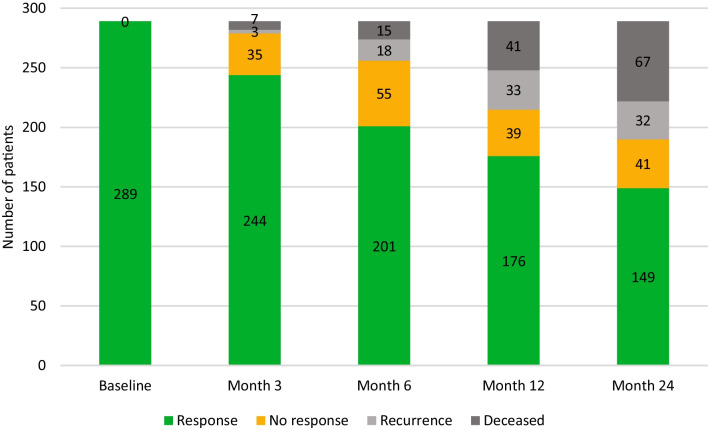
Response and non-response to questionnaires, recurrence and death among patients

During the study period, 369 patients with mucosal, salivary gland and cutaneous malignancies in the head and neck area were included in OncoLifeS. After exclusion of patients receiving palliative or non-standard treatment and patients not responding to baseline questionnaires, 289 patients remained in the study for analysis (Fig. 1). The mean age was 68.4 years and 68.5% were male. 54.5% of patients had advanced stage disease. Recurrence, death and response to follow-up questionnaires are shown in Fig. 2. From all patient and study characteristics, age [OR 3.21 (1.80–5.72)], time [per year OR 1.47 (1.10–1.97)] and subsequent death [OR 2.84 (1.62–4.99)] were significantly associated with non-response to follow-up questionnaires, in univariate GLLMs (Table 1). All remained significant in the multivariable model (Table 1).

**Table 1:** Patient characteristics and generalized linear mixed models for non-response

Patient characteristics	Value	Univariate models	*p *value	Multivariable model	*p *value
		OR (95%CI)		OR (95%CI)	
Age					
≤ 65 year	113 (39.1)	Ref.		Ref.	
> 65 year	176 (60.9)	**3.21 (1.80–5.72)**	**< 0.001**	**2.91 (1.61–5.28)**	**< 0.001**
Sex					
Male	198 (68.5)	Ref.			
Female	91 (31.5)	0.74 (0.41–1.31)	0.30		
Stage					
I–II	129 (45.4)	Ref.			
III–IV	155 (54.6)	0.36 (0.46–1.33)	0.36		
Primary treatment					
Surgery	163 (56.4)	Ref.			
Radiotherapy	84 (29.1)	0.51 (0.22–1.19)	0.12		
Chemotherapy	42 (14.5)	1.33 (0.74–2.40)	0.35		
Follow-up					
By mail	97 (33.5)	Ref.			
At outpatient clinic	192 (66.4)	0.86 (0.50–1.49)	0.59		
Time					
Per year		**1.47 (1.10–1.97)**	**0.009**	**1.70 (1.25–2.32)**	**0.001**
Subsequent recurrence					
No		Ref.			
Yes		1.64 (0.90–3.01)	0.11		
Subsequent death					
No		Ref.			
Yes		**2.84 (1.62–4.99)**	**< 0.001**	**3.13 (1.72–5.73)**	**< 0.001**

Generalized linear mixed models (binary logistic) showing odds ratios for non-response to follow-up questionnaires within the period of 24 months. Patients were excluded upward from recurrence or death. Values presented in *n* (%) unless otherwise specified. Bold values indicate significant findings (*p * < 0.05). OR = odds ratio; CI = confidence interval; ORL-HNS = otorhinolaryngology, head and neck surgery; RT = radiotherapy

Regarding GA items, restrictions in ADL [OR 4.46 (2.04–9.78)], IADL [OR 4.33 (2.27–8.24)], impaired mobility on the TUG [OR 3.95 (1.85–8.45)], signs of cognitive decline on the MMSE [OR 4.85 (2.28–10.35)], assisted living or living in a nursing home [OR 5.54 (2.63–11.67)] were significantly associated with non-response to questionnaires in univariate GLLMs (Table 2). This remained the case after adjusting for patient and study characteristics that showed significance in the univariate model, such as age, time and subsequent death (Table 2).

**Table 2:** Geriatric assessment, frailty screening and generalized linear mixed models for non-response

Geriatric assessment	Value	Univariate models	*p *value	Adjusted models*	*p *value
		OR (95%CI)		OR (95%CI)	
ACE-27					
None to mild (< 2)	164 (56.7)	Ref.		Ref.	
Moderate to severe (≥ 2)	125 (43.3)	1.21 (0.71–2.07)	0.48	0.82 (0.47–1.46)	0.51
Polypharmacy					
# medications (< 5)	188 (65.3)	Ref.		Ref.	
# medications (≥ 5)	100 (34.7)	1.04 (0.59–1.82)	0.90	0.70 (0.39–1.28)	0.25
MUST					
No malnutrition (0)	211 (78.1)	Ref.		Ref.	
Risk of malnutrition (≥ 1)	59 (21.9)	1.16 (0.59–2.31)	0.67	1.33 (− 0.64–2.77)	0.44
ADL					
No restrictions (0)	257 (88.9)	Ref.		Ref.	
Restrictions (≥ 1)	29 (10.1)	**4.46 (2.04–9.78)**	**< 0.001**	**3.16 (1.39–7.19)**	**0.006**
IADL					
No restrictions (0)	239 (83.0)	Ref.		Ref.	
Restrictions (≥ 1)	49 (17.0)	**4.33 (2.27–8.24)**	**< 0.001**	**3.11 (1.57–6.16)**	**0.001**
**TUG**					
< 13.5 s	242 (87.7)	Ref.		Ref.	
≥ 13.5 s	34 (12.3)	**3.95 (1.85–8.45)**	**< 0.001**	**2.60 (1.16–5.83)**	**0.02**
MMSE					
Normal cognition (> 24)	153 (88.2)	Ref.		Ref.	
Cognitive decline (≤ 24)	34 (11.8)	**4.85 (2.28–10.35)**	**< 0.001**	**3.57 (1.60–7.93)**	**0.002**
GDS-15					
No depression (< 6)	261 (91.3)	Ref.		Ref.	
Signs of depression (≥ 6)	25 (8.7)	0.84 (0.31–2.25)	0.73	0.68 (0.23–2.00)	0.48
Marital status					
In a relationship	216 (75.0)	Ref.		Ref.	
Single	72 (25.0)	1.56 (0.86–2.83)	0.14	1.42 (0.76–2.66)	0.28
Living situation					
Independent	253 (88.2)	Ref.		Ref.	
Requires help / nursing home	34 (11.8)	**5.54 (2.63–11.67)**	**< 0.001**	**3.83 (1.73–8.45)**	**0.001**
Educational level					
Lower education	119 (43.3)	Ref.		Ref.	
Middle or higher education	156 (56.7)	0.92 (0.63–1.59)	0.77	1.10 (0.63–1.94)	0.74
Frailty screening					
G8					
Non-frail (> 14)	126 (45.2)	Ref.		Ref.	
Frail (≤ 14)	153 (54.8)	**2.64 (1.51–4.59)**	**0.001**	**2.02 (1.12–2.66)**	**0.02**
GFI					
Non-frail (< 4)	203 (70.5)	Ref.		Ref.	
Frail (≥ 4)	85 (29.5)	**2.52 (1.44–4.44)**	**0.001**	**2.02 (1.11–3.68)**	**0.02**

Generalized linear mixed models (binary logistic) showing odds ratios for non-response to follow-up questionnaires within the period of 24 months. Patients were excluded upward from recurrence or death. Values presented in *n* (%) unless otherwise specified. *models were adjusted for age, time and subsequent death (items of the multivariable model in the right column of Table 1). Bold values indicate significant findings (*p * < 0.05). OR = odds ratio; CI = confidence interval; ACE-27 = Adult Comorbidity Evaluation-27; MUST = Malnutrition Universal Screening Tool; ADL = Activities of Daily Living; IADL = Instrumental Activities of Daily Living; TUG = Timed Up and Go; MMSE = Mini Mental State Examination; GDS-15 = Geriatric Depression Scale-15; G8 = Geriatric 8; GFI = Groningen Frailty Indicator

Frailty screening by both G8 and GFI, was significantly associated with non-response [OR respectively 2.64 (1.51–4.59) and 2.52 (1.44–4.44)], even after adjusting for the abovementioned factors (Table 2).

## Discussion

In this longitudinal observational study, we investigated whether frail patients exhibit more non-response to follow-up questionnaires than non-frail patients and whether specific items of a routinely performed GA are associated with this. Main findings were that frailty screening tools were associated with worse response to follow-up questionnaires. Besides, impaired ADL and IADL, restricted mobility, cognitive decline and dependent living situation were specifically associated with poorer response to follow-up questionnaires. These associations were independent of other significant factors, such as age, duration of the study and subsequent death during the study. To our knowledge, this is the first study demonstrating the association between geriatric factors and response to PROMs in patients with HNC. These results are important for the interpretation of all studies dealing with PROMS, because of the increasing proportion of older and frail patients.

In our study, higher age was significantly associated with non-response during follow-up. This is in line with some earlier studies [8, 28–30]; however, other studies found no significant differences [9, 31–33]. Comparison is difficult, given the different cancer types (and therewith age groups) and study methodologies which may explain the divergent outcomes.

A recent study, however, did investigate study retention and attrition in a longitudinal study of HNC patients in the Netherlands, collecting PROMs, fieldwork data and biobank materials up to 2 years [34]. In this study, age was not associated with attrition, unlike other factors such as higher tumour stage, poorer physical performance and worse comorbidity score. The latter, comorbidity, was in line with other studies [28, 30, 32]; however, not with our study which identified no significant differences in response between patients with none to mild and moderate to severe comorbidities. A reason for this may be the fact that other studies often assign patients with recurrent disease and even deceased patients to the attrition or non-response group as well. In this way, there is a risk of predicting death or recurrence rather than non-response due to other (geriatric) factors. In our study, we have excluded patients with recurrence or death from the analyses, from the moment that recurrence or death occurred. This gives superior understanding underlying non-response mechanisms.

Other items of GA or frailty screening with respect to non-response, drop-out or attrition have rarely been investigated and not at all in the unique population of HNC. In other studies, the most valuable data available is originating from the PROMs themselves that patients were asked to fill out, but then used at baseline as a predictor for drop-out. Among some different studies in other cohorts, poor functional status, symptom burden, depressive symptoms, cognitive failure, psychosocial symptoms, lower socioeconomic status, low educational level, and poor baseline QoL were associated with attrition [9, 29, 31–33]. It must be noted that study methodology differed greatly between studies, and none of the studies specifically aimed HNC. Besides, one may question the ability of a QoL questionnaire subscale to diagnose, e.g. ‘cognitive failure’, often based on just a few questions, compared to specifically developed screening tools such as MMSE in the case of cognition. In our current study, where we have employed well-known and frequently used instruments for GA (and not subscales of the PROMs), we have seen consistent associations of restricted ADL and IADL, poor mobility, cognitive decline and dependent living situation with non-response.

Frailty screening tools, such as the G8 and GFI, were significantly associated with increased non-response as well, which was also expected given the share of functional, cognitive and psychosocial items in the screening tools. This is in line with another study, in which frailty was significantly associated with drop-out from a cohort study [35].

Attrition is common in longitudinal studies, especially with the use of PROMs. When data are missing (completely) at random, this usually does not lead to bias. However, when attrition rates are distinct for different study groups, e.g. in this case when comparing frail to non-frail patients, this may introduce attrition bias [36]. Data may be not missing at random anymore, as for instance frail patients systematically respond worse to the questionnaires and may have different outcomes as well. In such studies, such as in studies evaluating QoL outcomes between frail and non-frail patients [37, 38], the observed differences may be an underestimation of the real difference. Although ideally this should be prevented ahead of time by creating a strategy to take care of frail patients at risk for dropping-out (e.g. alternative study visits, using patients peer support, supportive telephone contacts) [39], it is important to know how to handle and interpret these data. According to experts, mixed models remain the best choice for the analysis of repeated measures and longitudinal data [36].

Strengths of this study include the prospective inclusion of patients, the large range of validated screening instruments, the ability to adjust for relevant covariates such as subsequent death or recurrence and study characteristics, and the maximum use of data points using mixed models and therewith limiting bias as much as possible. Limitations of this study may be the different collection methods of PROMs between departments (which was adjusted for), the absence of information why patients dropped out, the relatively small and heterogeneous study cohort, which included both mucosal as cutaneous malignancies. Besides, by excluding patient not responding to baseline questionnaires, some form of bias may already be present from the beginning.

## Conclusion

Frailty, measured by deficiencies on GA, such as impaired ADL and IADL, restricted mobility, cognitive decline and dependent living situation, or by frailty screening instruments (G8 and GFI), is significantly associated with worse response to follow-up PROMs. This is of importance when handling and interpreting data on older or frail HNC patients, as with the resulting attrition bias the observed effects may be an underestimation of the real differences. Not only researchers but also clinicians need to be aware of this potential bias during the interpretation of studies dealing with PROMs, as the frailest patients are less likely to be included and more likely to be lost from follow-up.
